# Cumulative ecological risk and academic adjustment in freshmen: mediation by problematic mobile phone use and moderation by self-control

**DOI:** 10.3389/fpsyg.2025.1554500

**Published:** 2025-06-13

**Authors:** Meiqi Yu, Yanyu Xia, Weihao Ye, Jinjun Zhang, Yanan Gu

**Affiliations:** ^1^Center for Mental Health Development, China Jiliang University College of Modern Science and Technology, Zhejiang, China; ^2^Pinghu Normal College, Jiaxing University, Zhejiang, China; ^3^Department of Psychology, Zhejiang Normal University, Zhejiang, China; ^4^Mental Health Center, Harbin Huade University, Heilongjiang, China

**Keywords:** cumulative ecological risk, academic adjustment, problematic mobile phone use, self-control, freshmen

## Abstract

**Introduction:**

Previous research has shown that multiple ecological risks, including family, school, peer, and societal risk factors, significantly affect students' academic adjustment. Based on Ecological systems theory, the present study explored the effect of cumulative ecological risk (CER) on freshmen's academic adjustment, the mediating role of problematic mobile phone use (PMPU), and the moderating role of self-control.

**Methods:**

A total of 2,962 freshmen, of which 1,564 were male (52.80%), participated in this study and completed the Cumulative Ecological Risk Questionnaire, Chinese Version of the College Students' Problematic Mobile Network Usage Behavior Scale, Self-Control Scale, and Chinese Version of the Undergraduate's Learning Adjustment Test.

**Results:**

The results showed that (1) there were significant correlations between CER, PMPU, self-control, and academic adjustment; (2) CER significantly negatively predicted freshmen's academic adjustment; (3) PMPU had a mediating effect between CER and academic adjustment; and (4) self-control moderated the effects of CER on both PMPU and academic adjustment.

**Discussion:**

These results provide a theoretical and empirical basis for formulating appropriate countermeasures to enhance the academic adjustment of freshmen who face various ecological risks.

## 1 Introduction

College is a different learning environment in which freshmen face a multifaceted set of challenges that extend across academic, personal-emotional, institutional, and social domains (Hang and Guo, 2025). While this situation can be stressful and cause various psychological problems such as anxiety, depression, and loneliness (Li and Sun, 2023; Lindell et al., 2020), academic adjustment can help freshmen overcome these challenges. Academic adjustment refers to the dynamic adjustment of students' learning motivation, methods, and engagement according to the surrounding environment and changes in learning need to establish an optimal psychological and behavioral balance state conducive to academic success (Baker and Siryk, 1984). This study operationally measured academic adjustment using a standardized psychological scale that captures shifts in learning motivation, strategies, and engagement in response to academic demands. Academic adjustment is a key determinant of college students' academic success (Hassan et al., 2023). Previous research has found that freshmen tend to experience more academic difficulties and challenges in their first few months on campus (Elias, 2009). Thus, paying attention to students' academic adjustment is of great significance in improving the quality of higher education. Exploring the risk factors and mechanisms of freshmen's academic adjustment can provide an empirical reference for promoting academic education in this group scientifically.

### 1.1 The relationship between cumulative ecological risk and academic adjustment

The biological ecological model argues that individual development is simultaneously influenced by different, closely linked, and interacting ecological microsystems (Bronfenbrenner and Morris, 1998). This means that there is no single risk factor that plays a decisive role in problem behaviors. Such multiple risks may exhibit complex interactions and superposition effects (Evans et al., 2013). Ecological risk is an environmental risk that increases vulnerability to negative outcomes (Goldstein and Brooks, 2012). A previous study indicated that college students face high ecological risks from family, school, peers, and society (Guan et al., 2023), and a higher cumulative ecological risk (CER) can lead to greater adjustment difficulties (Appleyard et al., 2005).

Family risk was proven to be a key factor affecting college students' academic adjustment. One study showed that students with coherent and adaptable family systems had greater academic adjustment (Basharpoor et al., 2022). Family cohesion can help youths adapt to social and environmental challenges (Morris et al., 2007). Moreover, poverty/economic difficulties (Papageorgiou and Callaghan, 2020), poor parent-child relationships (Geng et al., 2023), attachment insecurity (Yang et al., 2024), and interparental conflict (Parsa et al., 2014) are all family ecological risk factors for academic adjustment.

As an essential part of students' daily academic lives, schools can also influence their academic adjustment. For instance, teachers' social support can improve students' academic outcomes (Tennant et al., 2015). Li (2022) confirmed that good teacher-student relationships can facilitate adaptive academic behavior among freshmen. In addition, students with a greater sense of belonging to school perform better in academic work (Gowing and Jackson, 2016). School connectedness has proven to be a significant predictor of freshmen's academic performance (Pang and Qiao, 2023).

Peer relationships are among the most important social relationships among college students (Zhou et al., 2023). Therefore, peer risk factors are important predictors of academic adjustment. For instance, college students who perceived peer support reported more adaptive academic achievements such as learning engagement (Shao and Kang, 2022), academic competence (Worley et al., 2023), and learning motivation (Marley and Wilcox, 2021). Additionally, adolescents are at an increased risk of externalizing problem behaviors when affiliated with deviant peers (Tien et al., 2019), which can lead to academic maladjustment.

Community is also a risk source that cannot be ignored. Exposure to community violence leads to lower academic performance, whereas increased perceived safety improves students' academic achievement (Milam et al., 2010). College students with higher perceived community security report a lower rate of academic challenges (Gezinski et al., 2024), while good neighborhood relations can promote their academic performance (Witherspoon et al., 2018).

Ecological risks from families, schools, peers, and the community always interact with each other. For instance, attachment theory holds that one's early attachment to one's parents forms an “internal working model” of themselves and others, which could potentially guide the establishment of future relationships between them and others (e.g., relatives, teachers, or peers) (Bowlby, 1982). Previous research has found that individuals with insecure attachment experience difficulty establishing peer relationships (Buote et al., 2009; Su et al., 2018). A study of medical postgraduates found that a warm parenting style can help build good teacher-student relationships (Chen and Li, 2024). In addition, conflict in parent-child relationships can put the child at risk of peer victimization, and poor teacher-student relationships might further aggravate this risk (Huang et al., 2024). Moreover, positive perceptions of community functioning have proven to be strongly correlated with better family functioning. For example, strong community ties can encourage parents to adopt positive parent-child communication (Byrnes and Miller, 2012), while neighborhood residential instability can negatively affect the quality of parent-child relationships (Riina et al., 2016). Therefore, when facing risks from a microsystem, one may also be exposed to the risks from other microsystems.

Previous studies have examined the risk factors for academic adjustment in different ecological settings, but focusing on a single ecological risk factor cannot accurately reflect the influencing mechanism of college students' academic adjustment. College students face more than one ecological risk factor in their daily lives, and various risk factors have synergistic effects in reality (Evans et al., 2013). This implies that additive risks could cause students to face more serious academic adjustment difficulties. Therefore, this study investigates the effects of CER on freshmen's academic adjustment and the underlying mechanisms using a cumulative risk model.

### 1.2 The mediating role of problematic mobile Internet use

Previous studies have found that ample leisure time, respite from academic pressure, curiosity, and excitement about novelty can cause freshmen to lose control and spend more time on smartphone activities associated with addiction (Zhang and Peng, 2022). Problematic mobile phone use (PMPU) refers to phone use characterized by a range of addictive symptoms associated with smartphone use, such as craving, dependence, tolerance, withdrawal, and negative consequences (Bianchi and Phillips, 2005). PMPU may have a mediating effect between CER and college students' academic adjustment. First, according to the psychological decompensation theory, negative experiences can hinder an individual's development and lead them to escape reality and engage in pathological Internet use (Wu et al., 2019), which can have negative consequences. Compared to non-addicted students, Internet-addicted students report more negative consequences in their daily academic studies (Chou and Hsiao, 2000). The arousal theory of motivation posits that Internet overuse may lead to a disorder in psychological arousal, resulting in a decline in motivation (Dong et al., 2011), attention (Hou et al., 2019), and academic performance (Masood et al., 2020). In addition, pathological Internet use leads to academic failure by impeding student adaptation (Díaz López et al., 2022; Wang and Zhang, 2024).

Second, existing research has demonstrated the impact of the CER on PMPU. For instance, good family functioning can reduce students' mobile phone dependence (Li et al., 2023; Hernandez et al., 2024). Moreover, a high sense of school belonging can reduce students' frequency of mobile phone use (Zhu et al., 2019). Previous studies have found that negative teacher–student and peer relationships reduce students' sense of school belonging, which, in turn, leads to more disinhibition and problem behaviors, including smartphone dependence (Bae, 2022). Adolescents with weak school bonds and low support from peers and teachers are more susceptible to problematic Internet use (Qi et al., 2020; Roser et al., 2016). Additionally, college students with low subjective social support tend to overuse their mobile phones (Haberlin and Atkin, 2022; Liu et al., 2024a).

Finally, PMPU may mediate the relationship between CER and academic adjustment. Support resources from families, schools, peers, and society are key to students' healthy growth. If the above domains include many risks, individuals will not find a necessary “comfortable place” in the real world, which will encourage their search for satisfaction in the smartphone network world (Tudorel, 2023). They may then spend excessive time online while faltering in their work or academic performance (Gull and Sravani, 2024; Yuan et al., 2011).

### 1.3 The moderating role of self-control

As an ability to alter one's own thoughts, feelings, or behaviors to achieve a specific goal (Baumeister et al., 1994), self-control potentially moderates the effect of CER on academic adjustment. Fewer constraints and more temptation in the environment require college freshmen to have sufficient self-control to cope. To achieve academic success, students must control behaviors that can interfere with their studies (Stork et al., 2016). Good self-control has been shown to help students achieve successful academic performance (Seibert et al., 2016). In addition, individuals with poor self-control may act recklessly and adopt maladaptive coping styles when experiencing adverse stress (Liu et al., 2024b); that is, they are more likely to experience academic adjustment difficulties (Externbrink et al., 2019; Shu et al., 2024).

Self-control may moderate the relationship between the CER and PMPU. The loss compensation hypothesis proposes that, when facing medium and high risks (including family, school, peer, and social risks), college students tend to seek psychological compensation on the Internet (Kardefelt-Winther, 2014). Over time, they may experience excessive Internet use. The protective-stabilizing model proposes that the relationship between risks and problems in adolescents' psychological development can be weakened by protective factors (Fergus and Zimmerman, 2005). Self-control is a process in which people try to combat inappropriate behavior and thinking patterns according to their needs and desires and establish appropriate behavior and thinking patterns (He et al., 2012). Therefore, it can be regarded as a protective psychological factor when facing risk. For example, people with high self-control abilities can reduce the frequency of checking on social networking sites (Cao et al., 2018; Kim et al., 2017), that is, higher self-control is more likely to reduce the chance of excessive Internet usage patterns than lower self-control.

However, Ego depletion theory holds that self-control strength or capacity is a limited internal resource (Baumeister, 2014). This resource is consumed when one attempts to control one's emotions, thoughts, or behaviors. Specifically, when self-control resources are depleted by prior needs, subsequent self-control attempts weaken (Baumeister et al., 1998). That is, for individuals with strong self-control abilities, coping with risk factors and resisting bad behavior (e.g., Internet addiction) may consume substantial amounts of self-control resources, which may in turn affect their academic performance. In addition, the strength model of self-regulation proposes that self-control requires selective allocation of limited resources. When the brain detects that limited resources are being consumed at an unsustainable rate, it tends to store more resources to resist overconsumption (Baumeister and Vohs, 2016). When people with strong self-control abilities face high risks, they recognize that they are in an energy crisis of self-consumption, which forces them to store resources. They will then adjust themselves to a “low power consumption” mode; as a result, they are likely to show large fluctuations in academic performance.

**Figure 1:**
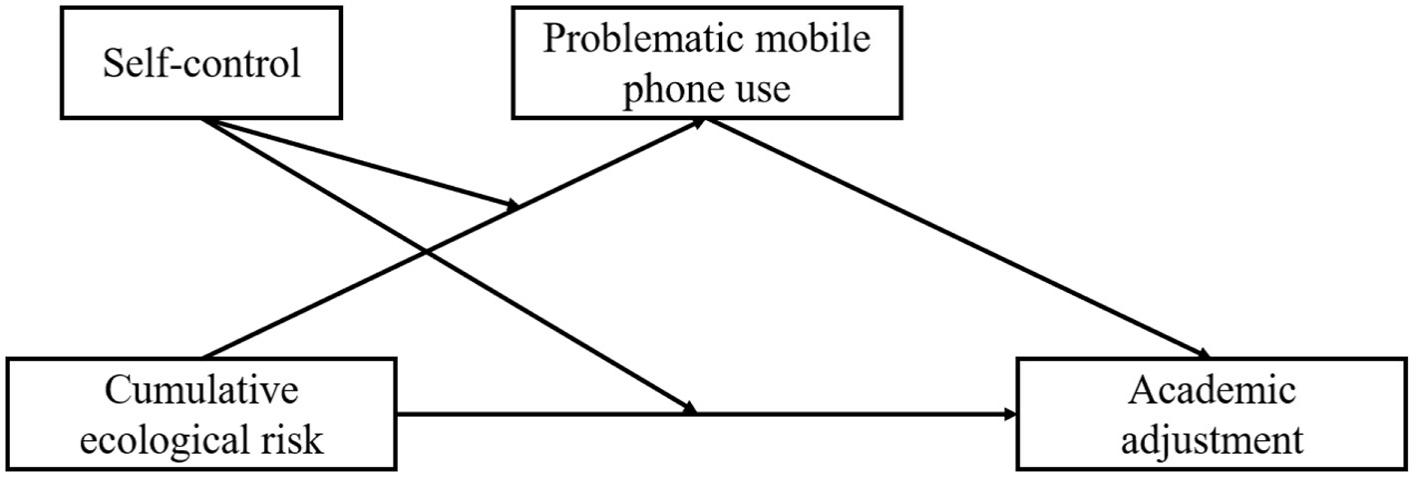
Conceptual framework.

In conclusion, to explore the risk factors of freshmen's academic adjustment and the underlying mechanism, this study proposes a moderated mediation model to test the mediating effect of PMPU between CER and academic adjustment, as well as the moderating role of self-control (see Figure 1) to provide empirical reference for the academic education of freshmen.

## 2 Materials and methods

### 2.1 Participants

A large online survey was conducted among freshmen from several universities in northeastern, northwestern, southwestern, central, and southeastern China. The survey was organized and distributed by college psychology counselors. The participants had the right to withdraw freely during the testing period. After removing invalid responses—defined as those with a “completion time lower than 3 min” and “wrong answer to the lie detector question”—a total of 2,962 freshmen (valid response rate: 92.77%) were included in the final analysis. Among them, 1,564 (52.8%) were male and 1,398 (47.2%) were female. Regarding university type, 746 participants (25.19%) were enrolled in first-tier public undergraduate universities, 1,420 (47.94%) were freshmen from second-tier public undergraduate universities, and 796 (26.87%) were enrolled in second-tier private undergraduate universities. In terms of regional background, 1,377 (46.49%) were from China's highly developed regions (e.g., Zhejiang Province and Guangdong Province), 757 (24.54%) were from moderately developed regions (e.g., Hunan Province and Henan Province), and 858 (28.97%) were from less-developed regions (e.g., Gansu Province and Heilongjiang Province).

### 2.2 Procedure

This study was conducted in accordance with the Declaration of Helsinki, as revised in 1989, and was approved by the local university. The online survey was organized by each participating university and distributed to the students. During the study, all participants were guaranteed confidentiality, anonymity, and the right to withdraw, and were debriefed.

### 2.3 Measures

#### 2.3.1 Cumulative ecological risk

Although all risk factors for academic adjustment should be included, only the particularly important and representative risk factors were included for feasibility and necessity. Based on previous studies (Basharpoor et al., 2022; Brubacher and Silinda, 2021; Jensen et al., 2021), this study selected ten ecological risk factors in the family context (i.e., parental education level, family financial difficulties, parental relationship, parent-child relationship), school context (i.e., teacher support, school connectedness), peer context (i.e., peer support, deviant peer affiliation), and community context (i.e., community safety, neighborhood support) to construct a CER index. The risk of each indicator was divided into two categories (presence = 1, absence = 0; the 25th percentile was used to define risk; see Table 1) to determine whether an individual was exposed to such risks. Finally, the total score of the ten indicators was defined as the CER. Using this method, previous studies have explored the effects of cumulative risks on non-suicidal self-injury (Liu et al., 2024b), mobile phone addiction (Tian et al., 2023), and problem behaviors (Sun et al., 2021). The ten indicator measurements are detailed as follows:

**Table 1:** Introduction of indicator scores and risk definition criteria.

**Indicator**	**Subsystems**	**Risk definition criteria**
Parental education level	Family	Parental education level is junior high school or below
Family financial difficulties	Family	Above 25th percentile
Parental relationship	Family	Above 25th percentile
Parent-child relationship	Family	Below 25th percentile
Teacher support	School	Below 25th percentile
School connectedness	School	Below 25th percentile
Peer support	Peer	Below 25th percentile
Deviant peer affiliation	Peer	Above 25th percentile
Community safety	Community	Below 25th percentile
Neighborhood support	Community	Below 25th percentile

##### 2.3.1.1 Parental education level

One item was used to measure the participants' parental education levels, referring to a previous study (Liu et al., 2024b). The question was “What is your parents' educational background?” The item was scored on a 6-point scale, with 1 being “postgraduate,” 2 being “undergraduate,” 3 being “associate degree,” 4 being “senior high school,” 5 being “junior high school,” and 6 being “primary school or below”.

##### 2.3.1.2 Family financial difficulties

This study used the Chinese Version of the Family Financial Difficulties Scale (Wang et al., 2010) based on the Economic Strain Scale developed by Wadsworth and Compas (2002). The scale has four items (e.g., “My family doesn't have enough money to buy new clothes”) and is scored on a 5-point scale ranging from 1 (never) to 5 (always). Higher scores indicate a more difficult financial situation. The Cronbach's α for family financial difficulties was 0.88.

##### 2.3.1.3 Parental relationship

This study used two items to measure parental relationships with reference to a previous study (Xiao et al., 2022). One item is “How is your parents' relationship?” which is scored on a 5-point scale ranging from 1 (very good) to 5 (very bad). Another item is, “Do your father and mother often quarrel?” which was scored on a 5-point scale ranging from 1 (never) to 5 (always). Higher scores indicate a more strained parental relationship. The Cronbach's α for the parental relationship was.70.

##### 2.3.1.4 Parent-child relationship

This study used the Parent-Child Relationship Scale (Buchanan et al., 1991). The scale consists of two dimensions, father-child relationship and mother-child relationship, with 18 items (e.g., “How often does your father/mother tell you that he/she loves you?”) and is scored on a 5-point scale ranging from 1 (strongly inconsistent) to 5 (strongly consistent). Higher scores indicate a closer father-child/mother-child relationship. The Cronbach's α for the parent-child relationship was 0.96.

##### 2.3.1.5 Teacher support

The study used the “Teacher Support” subscale of the Perceived School Climate Scale (Jia et al., 2009). The scale has seven items (e.g., “The teachers care about me.”). It is scored on a 4-point scale ranging from 1 (never) to 4 (always). Higher scores indicate better teacher support. The Cronbach's α for teacher support was 0.87.

##### 2.3.1.6 School connectedness

The study used the Chinese Version of the School Connectedness Scale (Yu et al., 2011). The scale has ten items (e.g., “I am proud to belong to this school.”) and is scored on a 5-point scale ranging from 1 (strongly disagree) to 5 (strongly agree). Higher scores indicate better school connectedness. The Cronbach's α for school connectedness was 0.91.

##### 2.3.1.7 Peer support

The study used the Peer Intimate Relationship subscale of the Healthy Kids Resilience Assessment, revised by Li et al. (2008). The scale has three items (e.g., “My friends care about me”) and is scored on a 4-point scale ranging from 1 (strongly inconsistent) to 4 (strongly consistent). Higher scores indicate better peer support. The Cronbach's α for peer support was 0.92.

##### 2.3.1.8 Deviant peer affiliation

The study used the Deviant Peer Affiliation Scale (Li et al., 2013). The scale has eight items (e.g., “How many of your good friends smoke?”) and is scored on a 5-point scale ranging from 1 (none) to 5 (all). Higher scores indicate more deviant peer affiliation. The Cronbach's α for deviant peer affiliation was 0.82.

##### 2.3.1.9 Community safety

The study used one item to measure community safety, referring to a previous study (Gerard and Buehler, 2004). The item was “Do you think your neighborhood is safe?” The item is scored on a 4-point scale ranging from 1 (very unsafe) to 4 (very safe). Higher scores indicate a safer community.

##### 2.3.1.10 Neighborhood support

The study used two items (e.g., “Are you familiar with your neighborhood?”) developed by Dong and Lin (2011). The items are scored on a 4-point scale ranging from 1 (strongly disagree) to 4 (strongly agree). Higher scores indicate better neighborhood support. The Cronbach's α for neighborhood support was 0.83.

#### 2.3.2 Academic adjustment

This study used the Chinese version of the Undergraduate Learning Adjustment Test (Feng et al., 2006). This scale has shown good reliability and validity (Yu et al., 2024). The scale consists of 29 items (e.g., “I adapt to university study”). It is scored on a 5-point scale ranging from 1 (strongly inconsistent) to 5 (strongly consistent). Higher scores indicate greater academic adjustment. The Cronbach's α for the scale in this study was 0.91.

#### 2.3.3 Problematic mobile internet use

This study used the Chinese version of the College Students' Problematic Mobile Network Usage Behavior Scale (Jiang et al., 2017). This scale has shown good reliability and validity among Chinese university students (Yu et al., 2024). The scale consists of 16 items (e.g., “I find myself spending more and more time using my phone”). It is scored on a 5-point scale ranging from 1 (strongly inconsistent) to 5 (strongly consistent). Higher scores indicate more serious PMPU. The Cronbach's α for the scale in this study was 0.88.

#### 2.3.4 Self-control

This study used the Self-Control Scale (Tangney et al., 2004). It has shown good reliability and validity (Xie et al., 2024). The scale has 13 items (e.g., “I can control my brand”) and is scored on a 5-point scale ranging from 1 (strongly inconsistent) to 5 (strongly consistent). Higher scores indicate greater self-control ability. The Cronbach's α for the scale in this study was 0.76.

### 2.4 Data analyses

Data analyses were performed using the Statistical Package for the Social Sciences (SPSS 26.0) and the Analysis of Moment Structures (AMOS 26.0) software. First, Harman's single-factor test was performed to examine the common method variance. The means and standard deviations of the continuous variables were used to summarize the descriptive information of the sample. Pearson's correlations were used to assess associations among the variables. Our research aims, including additional interactions, introduce unnecessary complexity. We employed Model 8 in the PROCESS macro (Hayes, 2017) to test our moderated mediation hypotheses, where the moderator influences both the first stage of the mediation pathway and the direct path. In addition, the covariates (gender and age) in the model were controlled, and the study variables were standardized. Finally, a multigroup analysis was conducted to examine the differences in the moderated mediation effects between the two genders.

## 3 Results

### 3.1 Common method bias test

Because this study relied heavily on self-reported measurements, we conducted Harman's single-factor test to conduct an exploratory factor analysis on all items of the six questionnaires. The results showed 18 eigenvalues >1 and the variance explained by the first factor was 24.25% (< 40%). Thus, common method variance is not obvious in this study.

### 3.2 Descriptive statistics and correlation analysis

Descriptive statistics and correlation analyses are shown in Table 2. The results indicate that CER is significantly positively correlated with PMPU and significantly negatively correlated with self-control and academic adjustment. PMPU is significantly negatively correlated with self-control and academic adjustment. Self-control is significantly positively correlated with academic adjustment.

**Table 2:** Descriptive statistics and correlations among variables.

**Variables**	***M* ±*SD***	**1**	**2**	**3**	**4**	**5**	**6**
1. Gender	-	-					
2. Age	18.53 ± 0.95	-	-				
3. CER	3.70 ± 2.31	−0.06^**^	0.23^***^	-			
4. PMPU	46.95 ± 10.10	0.02	0.007	0.17^***^	-		
5. Self-control	41.11 ± 7.36	0.02	−0.12^***^	−0.39^***^	−0.56^***^	-	
6. Academic adjustment	95.65 ± 15.57	0.02	−0.18^***^	−0.52^***^	−0.50^***^	0.67^***^	-

N = 2,962; Gender (0 = boy, 1 = girl); ^*^represents significant. ^*^p < 0.05, ^**^p < 0.01, ^***^p < 0.001.

### 3.3 Test of the moderated mediation model

After the standardization of all continuous variables, Model 8 was used to conduct the moderated mediation test. The results are summarized in Table 3. A summary of the statistics for the mediation effect tests is presented in Table 4. Gender (β = 0.03, *p* < 0.05), age (β = −0.05, *p* < 0.01), CER (β = −0.04, *p* < 0.05), and the interaction terms between CER and self-control (β = −0.03, *p* < 0.05) all significantly predict freshmen's PMPU with a small effect (Cohen, 1988). Self-control (β = −0.58, *p* < 0.001) significantly predicts freshmen's PMPU with a large effect. The results show that self-control significantly moderates the relationship between CER and PMPU such that the negative predictive effect of CER on PMPU is stronger at higher levels of self-control. Age (β = −0.06, *p* < 0.001) significantly predicts freshmen's academic adjustment with a small effect. PMPU (β = −0.21, *p* < 0.001), and the interaction terms between CER and self-control (β = −0.10, *p* < 0.001) significantly predict freshmen's academic adjustment with a medium effect. CER (β = −0.30, *p* < 0.001), and self-control (β = 0.43, *p* < 0.001) significantly predict freshmen's academic adjustment, with a large effect. Moreover, the results show that self-control significantly moderates the direct effect of CER on academic adjustment, such that the negative predictive effect of CER on academic adjustment is stronger at higher levels of self-control. According to Preacher and Kelley's (2011) effect size criteria, the bootstrap results show that the direct path of the mediation model is significant at a high self-control level (ES = −0.40, 95% CI = [−0.43, −0.37]), representing a large effect. Furthermore, the direct path of the mediation model at a low self-control level (ES = −0.21, 95% CI = [−0.24, −0.17]) is also significant, representing a large effect. However, the indirect effect is only significant under the high self-control condition (ES = 0.02, 95% CI = [0.01, 0.03]), representing a medium effect, which suggests that only freshmen with high self-control can improve their academic adjustment by reducing PMPU in the face of cumulative ecological risks. Structural equation modeling was conducted using AMOS 26.0, and the results showed good model fit (χ^2^/*df* = 21.16, GFI = 0.98, AGFI = 0.94, RMSEA = 0.08).

**Table 3:** Testing the moderated mediation effect of CER on academic adjustment.

**Outcome variables**	**Independent variables**	***R***	***R^2^***	***F***	**β**	***t***	**95% CI**
PMPU	Gender	0.57	0.32	282.59	0.03	2.08^*^	[0.02, 0.06]
	Age				−0.05	−3.27^**^	[−0.08, −0.02]
	CER				−0.04	−2.42^*^	[−0.07, −0.01]
	Self-control				−0.58	−35.54^***^	[−0.62, −0.55]
	CER × self-control				−0.03	−2.36^*^	[−0.06, −0.01]
Academic adjustment	Gender	0.76	0.57	651.60	−0.001	−0.10	[−0.03,0.02]
	Age				−0.06	−4.43^***^	[−0.08, −0.03]
	CER				−0.30	−22.50^***^	[−0.33, −0.28]
	PMPU				−0.21	−14.27^***^	[−0.24, −0.18]
	Self-control				0.43	27.09^***^	[.39,0.46]
	CER × self-control				−0.10	−9.39^***^	[−0.12, −0.08]

**Table 4:** Bootstrap results for direct and indirect effects.

**Self-control**	**Effect**	***SE***	**95% CI**
**Direct effect**			
Low (1 *SD* below the mean)	−0.21	0.02	[−0.24, −0.17]
Moderate (mean value)	−0.30	0.01	[−0.33, −0.28]
High (1 *SD* above the mean)	−0.40	0.02	[−0.43, −0.37]
**Indirect effect**			
Low (1 *SD* below the mean)	0.002	0.01	[−0.01,0.01]
Moderate (mean value)	0.01	0.004	[0.01, 0.02]
High (1 *SD* above the mean)	0.02	0.01	[0.01, 0.03]

**Figure 2:**
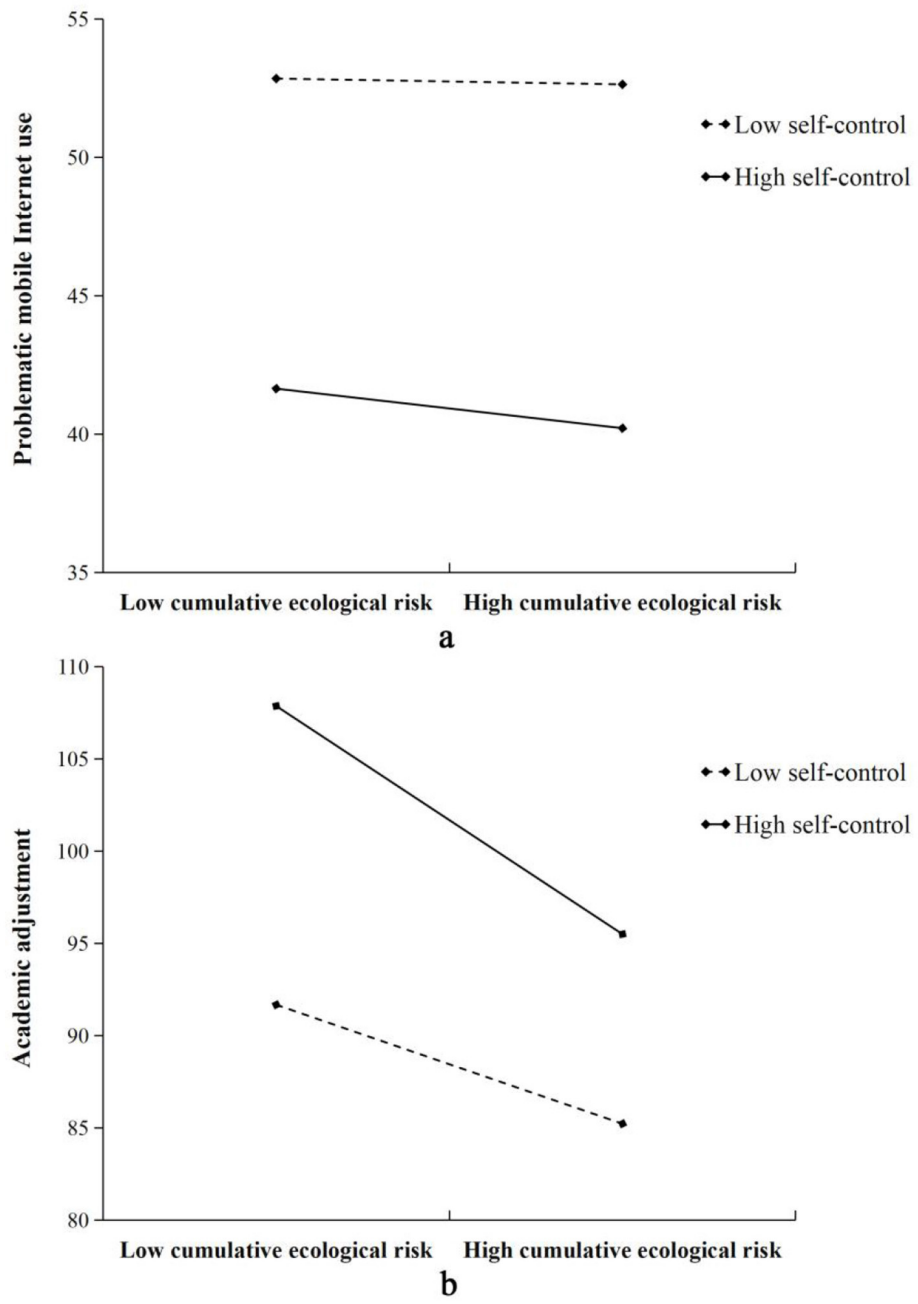
Moderating role of self-control. **(a)** The moderating role of self-control on the effect of CER on PMPU. **(b)** The moderating role of self-control on the effect of CER on academic adjustment.

To explain these moderating effects further, we conducted a simple slope test to examine the moderating role of self-control. As shown in Figure 2, the results in Figure 2a indicates that the predictive effect of CER on PMPU is not significant when self-control is low (β = −0.01, *p* > 0.05). However, the predictive effect of CER on PMPU is significantly negative when self-control is high (β = −0.07, *p* < 0.01), with a medium effect. In addition, the results in Figure 2b indicate that the predictive effect of CER on academic adjustment is largely negative and increases as self-control increases (β _low_= −0.21, *p* < 0.001; β _high_= −0.40, *p* < 0.001), representing large effects.

### 3.4 Multigroup analyses of the moderated mediation model according to gender

To test the applicability of the model to both genders, a multigroup analysis of structural invariance was established for both boys and girls to allow free estimation. As shown in Table 5, the observed good fit of the models for boys and girls indicates the feasibility of multigroup comparisons between structural equation models. The additional constraints imposed in the structural model resulted in a deterioration compared to the baseline model (Δχ^2^ = 55.5, *df* = 9, *p* < 0.001), which indicates a significant difference in the moderated mediating effects between boys and girls.

**Table 5:** Gender comparison of models.

**Model**	**χ^2^**	***df***	**χ^2^/*df***	**GFI**	**AGFI**	**CFI**	**RMSEA**	***Δχ*^2^(Δ*df)***	***p***
M_boys_	112.86	5	22.57	0.97	0.90	0.96	0.12		
M_girls_	179.44	5	35.89	0.96	0.82	0.91	0.16		
M_1Unconstrained_	298.26	11	27.11	0.97	0.87	0.94	0.09		
M_2Structuralweights_	353.75	20	17.69	0.96	0.92	0.93	0.07	55.5(9)	0.000

Therefore, we conducted multigroup analyses with gender group pairings to identify differences. The critical ratios (CRs) for the difference between groups with a z-score ≥ 1.96 were considered significantly different (Rong, 2009). As shown in Table 6, the effects of the interaction terms between CER and self-control (CR = 3.94, *p* < 0.05) on PMPU exhibit significant gender differences. Specifically, self-control significantly moderates the negative predictive effect of CER on boys' PMPU (β = −0.06, *p* < 0.001) with a small effect, while self–control does not moderate the effect of CER on girls' PMPU (β = 0.02, *p* = 0.30). Additionally, the effects of CER (CR = 3.94, *p* < 0.05), self–control (CR = −2.06, *p* < 0.05), and the interaction terms between CER and self–control (CR = −3.63, *p* < 0.05) on academic adjustment exhibit significant gender differences. Specifically, compared to girls (β = −0.24, *p* < 0.001), CER exerts a significantly stronger negative predictive effect on boys' academic adjustment (β = −0.35, *p* < 0.001) with a large effect. Compared to girls (β = 0.38, *p* < 0.001), self–control exerts a significantly stronger positive predictive effect on boys' academic adjustment (β = 0.44, *p* < 0.001) with a large effect. Further, compared to boys (β = – 0.08, *p* < 0.001), self–control moderates a larger negative predictive effect of CER on girls' academic adjustment (β = −0.16, *p* < 0.001) with a medium effect.

**Table 6:** Gender comparison of model path coefficients.

**Outcome variables**	**Independent variables**							

		**Boys**			**Girls**			
		β	***SE***	***p***	β	***SE***	***p***	**CR**
PMPU	CER	−0.03	0.02	0.30	−0.07	0.03	0.01	−1.32
	Self-control	−0.57	0.02	0.000	−0.60	0.03	0.000	−0.88
	CER × Self-control	−0.06	0.02	0.000	0.02	0.02	0.30	2.87^*^
Academic adjustment	CER	−0.35	0.02	0.000	−0.24	0.02	0.000	3.94^*^
	PMPU	−0.21	0.02	0.000	−0.21	0.02	0.000	0.12
	Self-control	0.44	0.02	0.000	0.38	0.02	0.000	−2.06^*^
	CER × Self-control	−0.08	0.01	0.000	−0.16	0.02	0.000	−3.63^*^

^*^p < 0.05.

## 4 Discussion

Freshmen's academic adjustment is affected by different ecosystems (Evans et al., 2013). However, previous studies have only focused on the impact of a single or a few ecological risk factors on academic adjustment (Gowing and Jackson, 2016; Shao and Kang, 2022; Yang et al., 2024). In contrast, this study investigated the effect of CER on academic adjustment and its underlying mechanism by selecting typical and representative risk factors related to students' academic adjustment and then constructing a CER index.

First, we found that CER negatively predicted freshmen's academic adjustment, which is consistent with previous findings (Appleyard et al., 2005; Geng et al., 2023; Shao and Kang, 2022). The ecosystem theory proposes that the academic adjustment of students is influenced by multiple ecological contexts, including family, school, peers, and society (Guan et al., 2023). In reality, these factors rarely occur in isolation (Flouri and Kallis, 2007). When exposed to a certain number of risk factors, the coping strategies employed by students become overwhelmed and subsequently exhausted (Ashworth and Humphrey, 2020), thereby increasing their vulnerability to negative academic outcomes (Goldstein and Brooks, 2012). Thus, universities should pay sufficient attention to and help freshmen who live in different adverse ecological environments (e.g., family poverty, parental alienation, parent-child conflict, peer rejection, and neighborhood conflict) for long periods.

In addition, the results indicated that PMPU mediated the relationship between CER and freshmen's academic adjustment and that this effect was moderated by self-control. These results are consistent with those of a previous research (Cao et al., 2018; Kim et al., 2017; Ma et al., 2023; Wang and Zhang, 2024), which showed that CER affects the academic adjustment of freshmen with high self-control regarding PMPU. Specifically, when faced with increased cumulative ecological risks, they mitigate decreased academic adjustment by reducing problematic mobile network use. However, PMPU could not mediate the effect of CER on the academic adjustment of freshmen with low self-control. Students' vulnerability increases under the stimulus of multiple risks, making them more prone to psychological problems (Kardefelt-Winther, 2014). This leads them to seek maladaptive psychological compensation through the Internet and to become addicted to it. Kandemir (2014) found that academic failure may be caused by procrastination in academic schedules and activities due to overindulgence in Internet usage. Excessive mobile phone use may also induce cognitive distraction (Masood et al., 2020) and procrastination (Geng et al., 2018), thus, negatively influencing students' academic performance. Fortunately, good self-control can help freshmen resist the temptation of excessive Internet usage and ease the pressure of academic adjustment.

Additionally, a simple slope analysis showed that the predictive effect of CER on PMPU was not significant when self-control was low; however, CER negatively predicted PMPU when self-control was high. Additionally, the predictive effect of CER on academic adjustment was largely negative and the slope increased as self-control increased. These results suggest that when encountering multiple ecological risks, freshmen with strong self-control are less prone to excessive mobile Internet use, but their academic adjustment is strongly affected. The ego depletion theory proposes that all self-control behaviors depend on a common resource (Baumeister, 2014). Once self-control resources are exhausted, future acts of self-control are impaired. Previous studies have shown that individuals must expend self-control resources to counter the adverse effects of external risks (Namusoke et al., 2024). People with high self-control abilities strictly control the use of mobile phones when facing high ecological risks, resulting in the excessive consumption of self-control resources. That is, freshmen with high self-control abilities may not have enough resources to control themselves and concentrate on academics. Conversely, the theory of resilience holds that the factors (e.g., self-control) that can help people adjust well in high-risk environments are referred to as promoting or protective factors (Masten, 2001). However, high demands for self-control increase limited resource consumption and make individuals conservative in resource allocation (Baumeister and Vohs, 2016), which causes drastic fluctuations in their academic performance when facing high risks.

We found differences in the effects of CER on academic adjustment between boys and girls. When faced with a high ecological risk, self-control was more helpful for boys. This was manifested by reduced dependence on problematic mobile use. Moreover, high ecological risk caused boys to face more severe academic adjustment difficulties, but self-control helped them adapt better to studying. Previous studies have found a stronger negative correlation between self-control and Internet addiction in men than in women (Li et al., 2021). In addition, boys appeared to be less self-controlled than girls (Duckworth et al., 2015), which leads to poor academic performance (Kingdon et al., 2017; Wu et al., 2016). Furthermore, when faced with high ecological risks, high self-control puts girls at a more significant fluctuation in academic adjustment than boys do. This may be because Asian girls are more accustomed to self-control (Wu et al., 2016), which leads to excessive consumption (Baumeister et al., 1998) or preservation (Baumeister and Vohs, 2016) of self-control resources when they face high risks; thus, they are unable to invest more resources in their studies and their academic adjustment is dramatically affected.

The main theoretical contribution of this study is its exploration of the cumulative effects of family, school, peer, and societal risk factors on freshmen's academic adjustment in China. Compared to previous research, our study highlights the synergistic effects among these risk factors, which improves research accuracy. This enriches relevant research on ecological systems theory and provides avenues and interpretive angles for improving the academic adjustment of freshmen.

Although many universities have implemented policies and measures to promote students' academic adjustment, there is still significant room for optimization and improvement in their coverage and implementation methods. Based on a university-as-ecosystem framework, our findings offer implications for policy reform and institutional optimization in higher education. Regarding risk screening, almost all colleges and universities investigate freshmen's family backgrounds and record them in the file system at the beginning of the semester. This is conducive to identifying the potential family related risk factors. However, the investigation of risk factors in freshmen's schools, peers, and community ecological subsystems is insufficient. Due to the additive effect of risk factors, it is necessary to comprehensively explore the potential risk factors from all four subsystems and compile a risk list, which can help schools screen high-risk groups for academic adjustment. Additionally, some universities investigate freshmen's academic adjustment during the first semester to identify students at a high risk of academic maladjustment and keep track of their academic performance in the following academic years. However, the ecological environment in this subsystem is constantly changing. Even if some students do not face risks during their first semester, they may encounter them later on. Therefore, colleges should consider the dynamic tracking of each student's risk factors to reduce the possibility of excessive mobile Internet use or academic maladjustment. Regarding policy restrictions, most colleges and universities adopt a relaxed policy regarding freshmen's use of mobile phones. However, given the mediation of problematic mobile phone use, colleges can restrict or control the use of mobile phones on special occasions as necessary. For instance, some colleges continue high school management policies in which students must hand on their mobile phones in class. This policy can force students to focus in class and increase their learning input; conversely, it can also reduce their dependence on mobile phones. Regarding faculty support, the university's current psychological faculty support system relies primarily on counselors conducting standardized monthly one-on-one talks with students to grasp changes in risk factors around students. However, the counselor's caseload and frequency of talk create a systemic vulnerability in risk surveillance. To address this issue, we propose the implementation of a multitiered monitoring framework involving course instructors, class teachers, and life teachers in systematic risk assessment protocols. As for academic structure, we observe the phenomenon of “valuing knowledge over feelings” in current college education, translated into the higher education overemphasis on cognitive development (e.g., 98% of curriculum hours) and neglect of students' psychological needs (e.g., only 2 credits of compulsory psychological health courses). Therefore, compulsory courses on psychological health should be emphasized, and optional psychological courses or group training should be actively set up to help students improve their psychological resilience to fight against risks. In relation to institutional culture, universities should foster a supportive psychological climate by promoting psychologically oriented student activities. This can be achieved by establishing peer-led psychological associations, organizing monthly thematic wellness campaigns, and conducting interactive psycho-educational workshops.

Additionally, compared to the strict management policy of Chinese high schools, a more liberal university policy is not conducive to cultivating freshmen's self-discipline consciousness. When students show addictive behaviors or poor academic performance, their counselors may verbally persuade them to strengthen their self-control of bad behaviors, which often shows a limited effect. Therefore, colleges should adopt systematic and scientific methods and policies to strengthen their self-control abilities. This can be achieved through initiatives, such as integrating relevant courses into the curriculum or offering group counseling programs. Our findings highlighted the need for gender-differentiated self-control training. Specifically, we showed that self-control can significantly reduce boys' PMPU, helping them better adapt to academic work. Notably, boys generally showed worse self-control than girls did, indicating that they may have insufficient potential self-control resources. Therefore, universities should focus on cultivating boys' self-control consciousness to promote the accumulation of self-control resources. Specifically, boys should be given targeted training in time management to help them rationally arrange their mobile device use time, quantify their learning objectives, and set a daily workload that must be completed and strictly implemented. Furthermore, our findings revealed that girls with high self-control showed inconsistent academic responses at a high risk. This may indicate maladaptive allocation of self-control resources, such that excessive self-regulation in some areas may inadvertently exacerbate academic stress. Notably, girls may show higher emotional sensitivity than boys when facing high risks. To manage these negative emotions, they need to expend many resources on self-control. Therefore, universities should focus on equipping girls with strategies to regulate the negative emotions arising from high-risk environments. Training programs should emphasize healthy emotional expression and promote adaptive coping mechanisms. By alleviating the burden of emotional self-control, universities can help optimize girls' overall self-control resource allocation.

This study has some limitations. First, it used a cross-sectional design, which limited our ability to discern the causal relationships between variables. Future longitudinal follow-up studies could help us gain better insights into causal relationships. Second, this study was conducted only among freshmen. Future research should encompass diverse groups of participants with different academic qualifications, grades, and other potential influencing factors to better explore the mechanism of CER on students' academic adjustment. For example, the lack of a comparative analysis of college students in urban and rural educational settings may limit the applicability of our findings, and thus should be considered in future studies. Third, the self-reported data in this study may be influenced by the effect of social expectations. To enhance objectivity and reliability, future research should incorporate data triangulation from multiple sources such as academic performance records (e.g., course grades), class engagement metrics (e.g., frequency of active participation in question-answering behaviors), and third-party evaluations (e.g., mobile phone overuse behavior feedback from teachers). Finally, this study collected data on a select number of typical and representative risk factors; therefore, future studies should include more potential risk factors (e.g., academic structure and institutional culture in the school risks aspect) and covariates (e.g., university environment and prior academic performance) to validate our results.

## 5 Conclusion

In summary, this study found that (1) CER negatively predicted freshmen's academic adjustment, (2) PMPU had a mediating effect between CER and freshmen's academic adjustment, and (3) self-control moderated the effects of CER on both PMPU and academic adjustment.

## Data Availability

The original contributions presented in the study are included in the article/supplementary material, further inquiries can be directed to the corresponding author.
